# Fatty acid metabolism influences the immune microenvironment in papillary thyroid cancer and identifies SCD as a novel biomarker

**DOI:** 10.3389/fendo.2025.1534393

**Published:** 2025-02-27

**Authors:** Bingbing Shen, Yu Zhang, Yan Tie

**Affiliations:** ^1^ Division of Thyroid Surgery, Department of General Surgery, Laboratory of Thyroid and Parathyroid Diseases, Frontiers Science Center for Disease-Related Molecular Network, West China Hospital, Sichuan University, Chengdu, China; ^2^ Laboratory of Aging Research and Cancer Drug Target, State Key Laboratory of Biotherapy and Cancer Center, National Clinical Research Center for Geriatrics, West China Hospital, Sichuan University, Chengdu, China; ^3^ Department of Biotherapy, Cancer Center, West China Hospital, Sichuan University, Chengdu, China

**Keywords:** papillary thyroid cancer, fatty acid metabolism, immune, bioinformatics, SCD

## Abstract

**Background:**

Papillary thyroid carcinoma (PTC) is a common endocrine tumor with a rapidly increasing incidence. While surgery and radioactive iodine treatment are effective for most patients, they impose significant economic and psychological burdens. Metabolic dysregulation, particularly in fatty acid metabolism (FAM), plays a critical role in cancer progression and immune responses. Identifying key FAM-related genes in PTC may provide valuable biomarkers and potential treatment candidates.

**Materials and methods:**

We analyzed 309 FAM-related genes to build a prognostic signature. DEGs were identified and a multivariate Cox regression model was utilized to establish a robust prognostic signature, which was validated by evaluating its associations with clinical features, immune responses, and tumor progression. Lastly, we examined the expression of key FAM-related genes in PTC cell lines and assessed that silencing *SCD* disturbs the proliferation, invasion, and migration of PTC cells.

**Results:**

We identified three key FAM-related genes, *ACACB*, *ADH1B*, and *SCD*, as significant prognostic markers. Immunological analysis uncovered that low-risk patients exhibited higher immune cell abundance and increased expression of immune checkpoints, indicating a better response to immunotherapy. In contrast, high-risk patients showed lower immune cell abundance and immune checkpoint expression, suggesting poorer immunotherapy outcomes. Experimental validation demonstrated that *ACACB* and *ADH1B* were downregulated, while *SCD* was upregulated in PTC cell lines. Furthermore, silencing *SCD* inhibited PTC cell proliferation, migration, and invasion.

**Conclusion:**

Our study underscores the pivotal role of FAM-related genes, particularly *ACACB*, *ADH1B*, and *SCD*, in the progression and immune regulation of PTC. The prognostic signature derived from these genes represents a valuable tool for predicting clinical outcomes and guiding personalized treatment strategies. Among these, *SCD* stands out as a promising therapeutic target for PTC, warranting further research to validate these findings and uncover its underlying molecular mechanisms.

## Introduction

Thyroid cancer is a prevalent endocrine malignancy, with papillary thyroid cancer (PTC) being the most common subtype, representing approximately 90% of all thyroid cancer (1). The incidence of thyroid cancer is rising rapidly worldwide in large part because of the increasing incidence of PTC (2). Although the prognosis of thyroid cancer patients is better than that of other malignant tumors, 47,485 patients still died from thyroid cancer in 2022 (3). So, it is vital to explore the key molecular markers that influence the progression of PTC.

Fatty acid metabolism (FAM) plays an important role in maintaining cellular homeostasis, as it is complexly involved in membrane synthesis, energy storage, and signal transduction (4). FAM upregulation may represent an adaptive response to the metabolic demands of tumor cells and is involved in multiple cellular processes, including cancer cell growth (5). In cancer cells, FAM upregulation not only provides the necessary lipids for membrane synthesis but also supports tumor initiation, progression, and resistance to therapy (6, 7). FAM reprogramming in cancer includes some key enzyme upregulation as well as enhanced fatty acid uptake and oxidation (4, 6, 8). These processes can promote tumor cell growth and proliferation by overcoming the lack of nutrients and a hypoxic environment (9). Recently, multiple studies have highlighted the significance of FAM in thyroid cancer, particularly PTC. Chu J. et al. found that *circPCNXL2* enhanced tumor cell growth by influencing the *de-novo* synthesis of fatty acids (10). Lu J. et al. demonstrated through multi-omics analyses that fatty acid metabolism, including hydrolysis, transport, and oxidation, is significantly upregulated in PTC (11). These studies provided novel directions for therapy by targeting dysregulated FAM-related genes in PTC. So, exploring the specific roles of FAM-related genes could provide valuable insights into disease mechanisms and novel biomarkers for the diagnosis, prognosis, and treatment of PTC.

**Figure 1:**
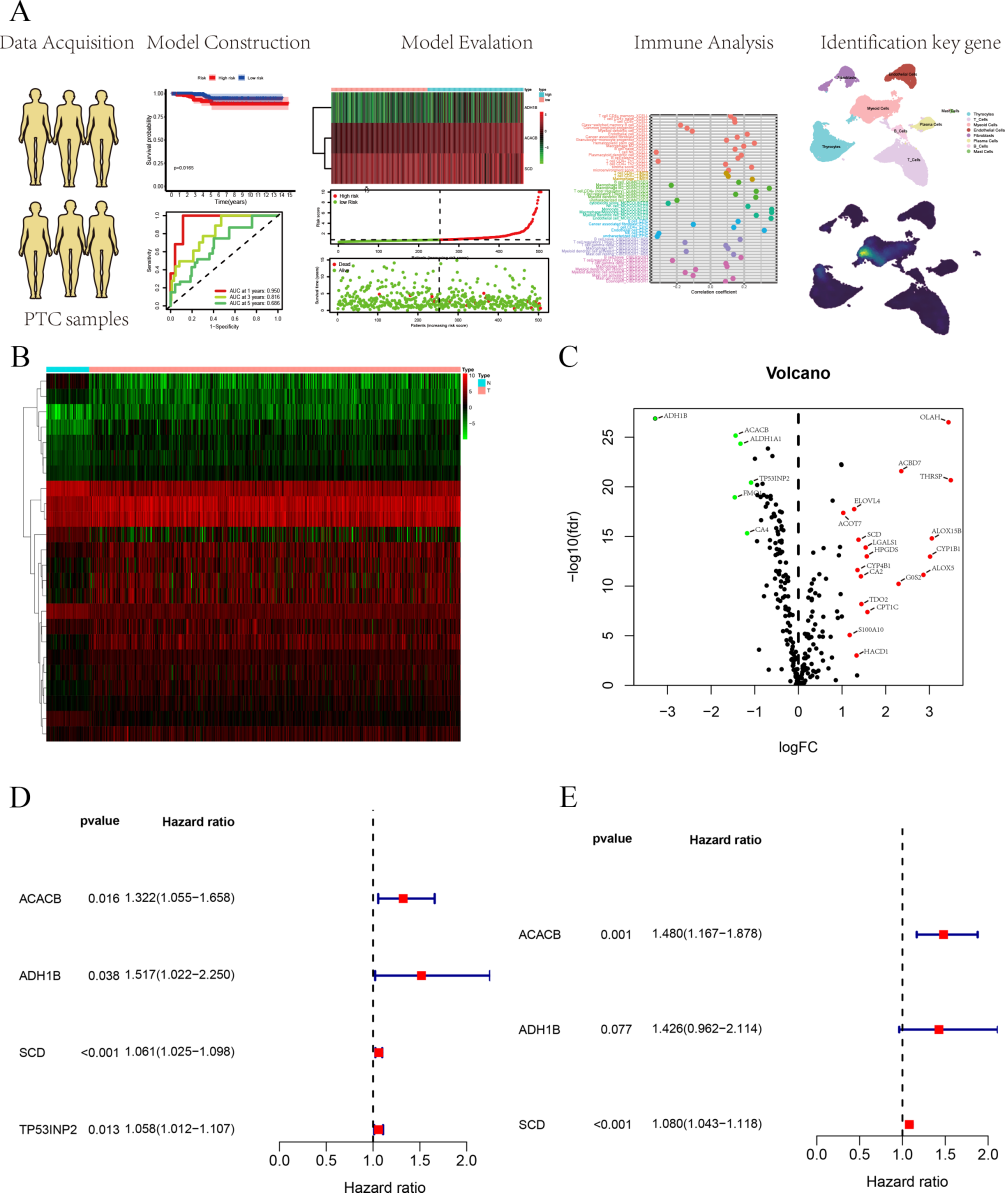
**(A)** Flowchart of this study. **(B)** The heatmap of DEGs based on FAM-related genes. **(C)** Volcano plot of DEGs based on FAM-related genes. **(D)** Univariate Cox regression analysis for survival-related genes. **(E)** Construction of the prognostic signature based on the three FAM-related genes using multivariate Cox regression analysis.

In this study, the FAM-related genes originated from the Molecular Signatures Database (MSigDB). Furthermore, we constructed a polygenic prognostic model based on a TCGA cohort for PTC. A comprehensive analysis was employed to evaluate the relationship between the prognostic model and immune and clinical features. Finally, we performed experiments to validate the impact of FAM-related genes on PTC progression. We concluded that further investigation is essential to elucidate the underlying mechanisms by which FAM-related genes influence PTC progression. The flowchart of this study is shown in **Figure 1A**.

## Materials and methods

### Data acquisition

FAM-related genes were sourced from the MSigDB available at https://www.gsea-msigdb.org/gsea/msigdb, a comprehensive resource that curates gene sets to facilitate functional enrichment analysis and the biological interpretation of genomic data (12–15). The Cancer Genome Atlas (TCGA) is a comprehensive database that integrates genomic and clinical data across various cancer types, serving as a valuable resource for cancer research (16). The TCGA cohort includes 59 normal thyroid tissues and 513 PTC tissues. We used survival, clinical features, and expression data in the TCGA cohort. Gene Expression Omnibus (GEO) is a public repository designed for the storage and sharing of gene expression and functional genomics data from high-throughput experiments. We used the GSE29265 and GSE33630 cohorts. The GSE29265 cohort includes 20 normal and 20 PTC tissues (17). The GSE33630 cohort includes 45 normal and 49 PTC tissues (18, 19).

The scRNA-seq data of GSE232237 were obtained from the GEO, which included three normal thyroid, seven PTC, and five anaplastic thyroid cancer (ATC) cases (20). In this study, we selected seven PTC samples to perform our analysis. The R package “Seruat” was utilized to perform the single-cell analysis. According to cell-specific markers, we isolated eight types of cells, namely, thyrocytes (“*TG*,” “*EPCAM*,” “*KRT18*”), T cells (“*CD3D*,” “*CD3E*,” “*CD8A*”), B cells (“*CD79A*,” “*MS4A1*,” “*IGHG1*”), myeloid cells (“*CD68*,” “*CD163*,” “*LYZ*”), endothelial cells (“*PECAM1*,” “*VWF*,” “*CLDN5*”), plasma cells (“*MZB1*,” “*SDC1*,” “*CD79A*”), mast cells (“*CST3*,” “*KIT*,” “*CPA3*”), and fibroblasts (“*FGF7*,” “*COL1A1*,” “*COL1A2*”).

### Construction and verification of the FAM-related gene prognostic signature

The RStudio (Version 4.4.1) software was used to construct the FAM-related gene prognostic signature. In this study, we employed several R packages, including “survival,” “survminer,” “heatmap,” “survivalROC,” “ComplexHeatmap,” “ggplot2,” “ggpubr,” “ggExtra,” and “limma.” To build the prognostic signature, the “survival” R package was employed for multivariate Cox regression analysis. Survival analysis utilized the “survival” and “survminer” R packages. Additionally, the “survival” R package was also used for both univariate and multivariate Cox regression analyses. A heatmap of three FAM-related genes was generated utilizing the “heatmap” R package. The ROC curve was plotted employing the “survivalROC” R package. The “ComplexHeatmap” R package was used to depict the association between clinical features and risk scores.

### Functional analysis and somatic mutation landscape

Gene set enrichment analysis (GSEA) is a computational method that evaluates whether predefined gene sets exhibit significant changes in expression across distinct biological states or conditions. In this study, the GSEA software was utilized to carry out function analysis. The plots for multiple GSEA were generated using the “plyr,” “ggplot2,” “grid,” and “gridExtra” R packages.

Mutation data were downloaded from the TCGA database. Mutation analysis mainly employed the “maftools” R package to generate waterfall plots, which visually represented the mutation frequency and types across samples, displaying gene-wise and sample-wise mutation distributions to highlight common mutations and patterns in genomic data.

### Immune-related analysis of the prognostic signature

The CIBERSORT algorithm, based on linear support vector regression (SVR), is a widely recognized and reliable machine learning technique for deconvolving expression matrices to evaluate the ratio of 22 distinct human immune cell subtypes (21). In this study, based on the CIBERSORT algorithm, we evaluated the immune cell abundance in each PTC sample from the TCGA database. In addition, we used two R packages: “preprocessCore” and “limma”.

Next, we utilized seven algorithms, namely, CIBERSORT-ABS (21), CIBERSORT (21), Tumor Immune Estimation Resource (TIMER) (22), QUANTISEQ (23), MCPcounter (24), xCell (25), and EPIC (26), to comprehensively analyze the immune landscape of two groups, which enabled us to assess various aspects of immune cell infiltration and characterize the immunological features in greater detail. We used the following eight R packages: “limma,” “scales,” “ggplot2,” “ggtext,” “reshape2,” “tidyverse,” “ggpubr,” and “pheatmap.” Additionally, immune function analysis was carried out utilizing five R packages: “limma,” “GSVA,” “GSEABase,” “ggpubr,” and “reshape2”.

### Cell culture and transfection

In this study, all cell lines were purchased from the China Type Culture Collection (CTCC). Nthy-ori 3-1 is a normal thyroid cell line, while TPC-1, K1, KTC-1, and BCPAP are PTC cell lines. Nthy-ori 3-1 cells were cultured in Dulbecco’s modified eagle medium (DMEM). TPC-1, KTC-1, and BCPAP cells were maintained in Roswell Park Memorial Institute 1640 (RPMI-1640) medium, and K1 cells were grown in DMEM/F12 (DMEM and Ham’s F-12 nutrient mixture) medium. All cells were grown in media with 10% fetal bovine serum (FBS) and 1% penicillin–streptomycin and incubated at 37°C with 5% CO_2_.

For the cell transfection of TPC-1, cell transfection was initiated when the cell density reached approximately 30%. Lipofectamine 2000 (Lip2000) was used as the transfection reagent. First, the optimized minimum essential medium was separately mixed with Lip2000 and siRNA, and each mixture was incubated for 5 min. The two solutions were then combined for an additional 15 minutes. The resulting transfection mixture was added to the complete RPMI-1640 medium without antibiotics. After 6–8 h, the medium was replaced, and *SCD* knockdown efficiency was evaluated 24 h post-transfection.

### Quantitative polymerase chain reaction

According to the manufacturer’s instructions, the TRIzol reagent was employed to acquire total ribonucleic acid). Next, complementary DNA (cDNA) was obtained by performing a reverse transcription assay. Lastly, qPCR was utilized to assess relative messenger ribonucleic acid (mRNA) expression. The 2^−ΔΔCt^ method was used to calculate relative expression. The primer names and primer sequences are shown in **Supplementary Table S1**.

### CCK8, colony formation, scratch, and Transwell assays

For the CCK8 assay, after successful transfection of TPC-1 cells, 3,000 cells from both the control and knockdown groups were cultured in 96-well plates. Cell viability was checked by incubating the cells for 0, 24, 48, and 72 h, and optical density (OD) at 450 nm was determined at each time point using a microplate reader.

For the scratch assay, after successful transfection of TPC-1 cells, cells were cultured until they reached 100%. A scratch was made using a 200-µL pipette tip, followed by PBS washing to remove the medium. Images were captured immediately after scratching and then again at 24 and 48 h to monitor wound healing.

For the colony formation assay, after successful transfection of TPC-1 cells, 1,000 cells from both the control and knockdown groups were seeded into six-well plates. The medium was replaced every 3–5 days. After 14 days, the plates were collected, washed with PBS, fixed with paraformaldehyde, and stained with crystal violet, and the images were taken after the plates dried using an Olympus microscope.

For the Transwell assay, after successful transfection of TPC-1 cells, 30,000 cells from both the control and knockdown groups were mixed with 260 µL of serum-free medium and transferred to the top chamber of the Transwell plates. The bottom chamber contained 750 µL of complete medium. For the invasion assay, 20% Matrigel was precoated on the bottom of the upper chamber, while for the migration assay, no Matrigel was used. After 24 h, the upper chamber was removed, washed, fixed, stained, and imaged using an Olympus microscope.

### Statistical analysis

RStudio (Version 4.4.1) and GraphPad Prism (Version 10.1.0) were utilized to perform statistical analysis. In this study, a *p*-value less than 0.05 was considered statistically significant.

## Results

### Construction of the prognostic signature for PTC patients

FAM-related genes were acquired from the MSigDB database (HALLMARK, KEGG, REACTOME), and 309 FAM-related genes were used for follow-up analyses (**Supplementary Table S2**). We analyzed DEGs from 309 FAM-related genes based on logFC more than 1 and *p*-value <0.05, in which 24 DEGs were identified and employed to build the prognostic signature (**Supplementary Table S3**). We used a heatmap and a volcano plot to display 25 DEGs associated with FAM (**Figures 1B, C**). Furthermore, we selected survival-related genes through univariate Cox regression analysis, and four FAM-related genes were selected (**Figure 1D**). Lastly, multivariate Cox regression analysis was utilized to build a three FAM-related gene prognostic signature (**Figure 1E**).

### Verification of the prognostic signature in the TCGA cohort

**Figure 2:**
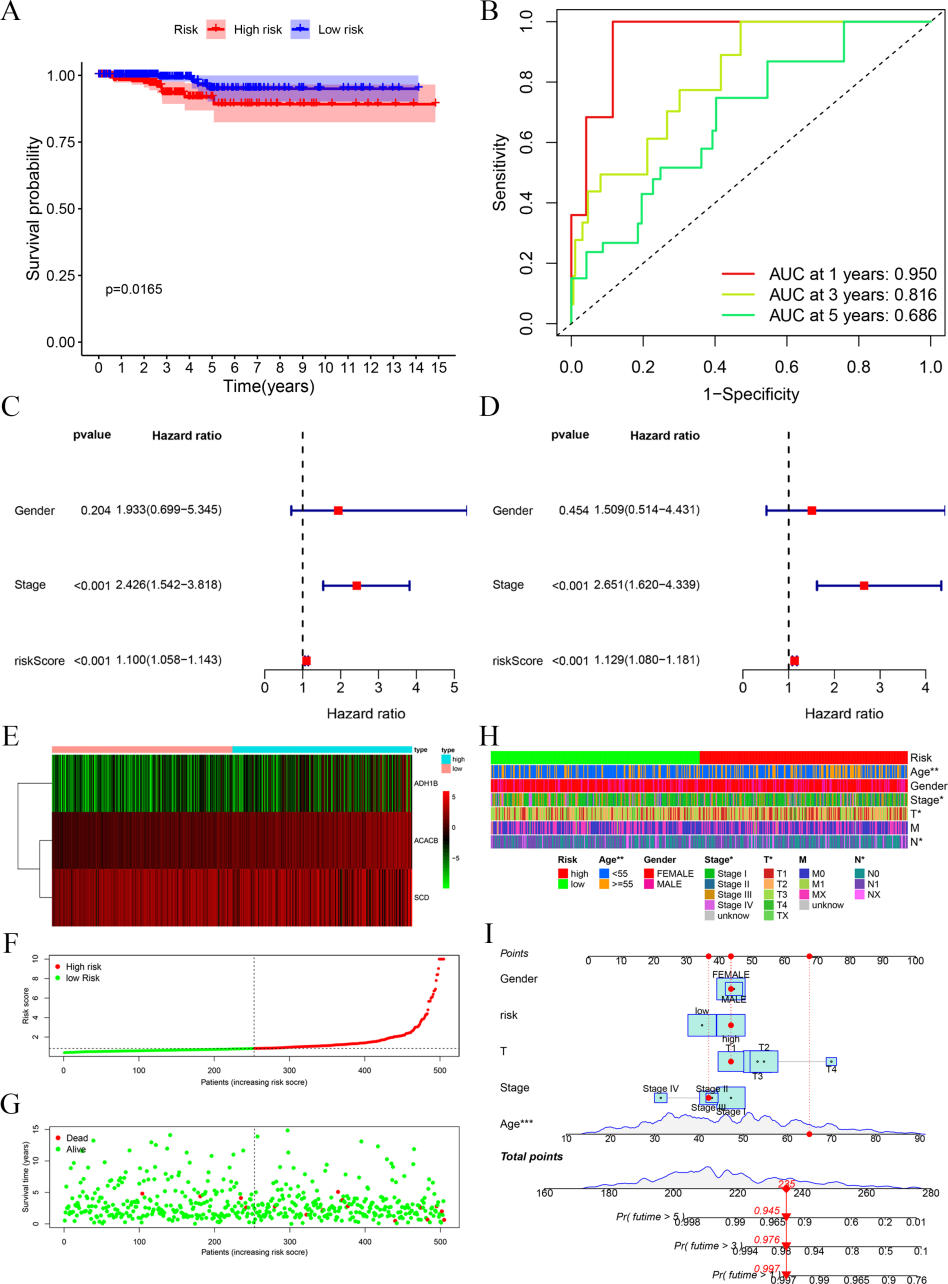
Evaluation of the prognostic signature. **(A)** Survival analysis of high and low risks showed that the high-risk group possessed a worse OS. **(B)** ROC curves at 1, 3, and 5 years showed the model has good predictive ability. **(C, D)** Univariate and multivariate Cox regression analyses of the prognostic signature identified risk score to possess a better independent prognostic effect. **(E)** Heatmap of the three FAM-related genes. **(F)** Risk score distribution for PTC patients. **(G)** Survival state curve for PTC patients. **(H)** The association between prognostic signature and clinical features, including age, gender, and TNM stage. **(I)** Development of the FAM clinicopathologic nomogram for predicting the 1-, 3-, and 5-year OS for PTC patients by incorporating risk score, age, gender, and TNM stage. *p < 0.05, **p < 0.01.

Through the above analyses, we successfully constructed the prognostic signature based on three FAM-related genes. It was observed through survival analysis that compared with the low-risk group, the high-risk group had a shorter survival probability (**Figure 2A**). The receiver operating characteristic (ROC) curve demonstrates that the prognostic signature exhibits high predictive efficacy, with area under the curve (AUC) values of 0.95 at 1 year, 0.816 at 2 years, and 0.686 at 3 years (**Figure 2B**). Univariate and multivariate regression analyses showed that stage and risk score are independent prognostic factors for PTC patients (**Figures 2C, D**). We depicted the heatmap of the three FAM-related genes in this risk group (**Figure 2E**). In addition, we also displayed the risk score curve (**Figure 2F**) and survival state distribution (**Figure 2G**), which confirmed the reasonable distribution between the two risk groups. Next, we integrated the clinical characteristics of PTC and analyzed their correlation with risk score, presenting the results in a heatmap for clarity (**Figure 2H**). The heatmap revealed that risk score is associated with age, T stage, and N stage. Lastly, a predictive nomogram was generated by integrating multiple clinical factors with risk scores (**Figure 2I**). These scores can be employed to estimate the survival probabilities of PTC patients.

### Functional analysis and somatic mutation analysis of the prognostic signature

**Figure 3:**
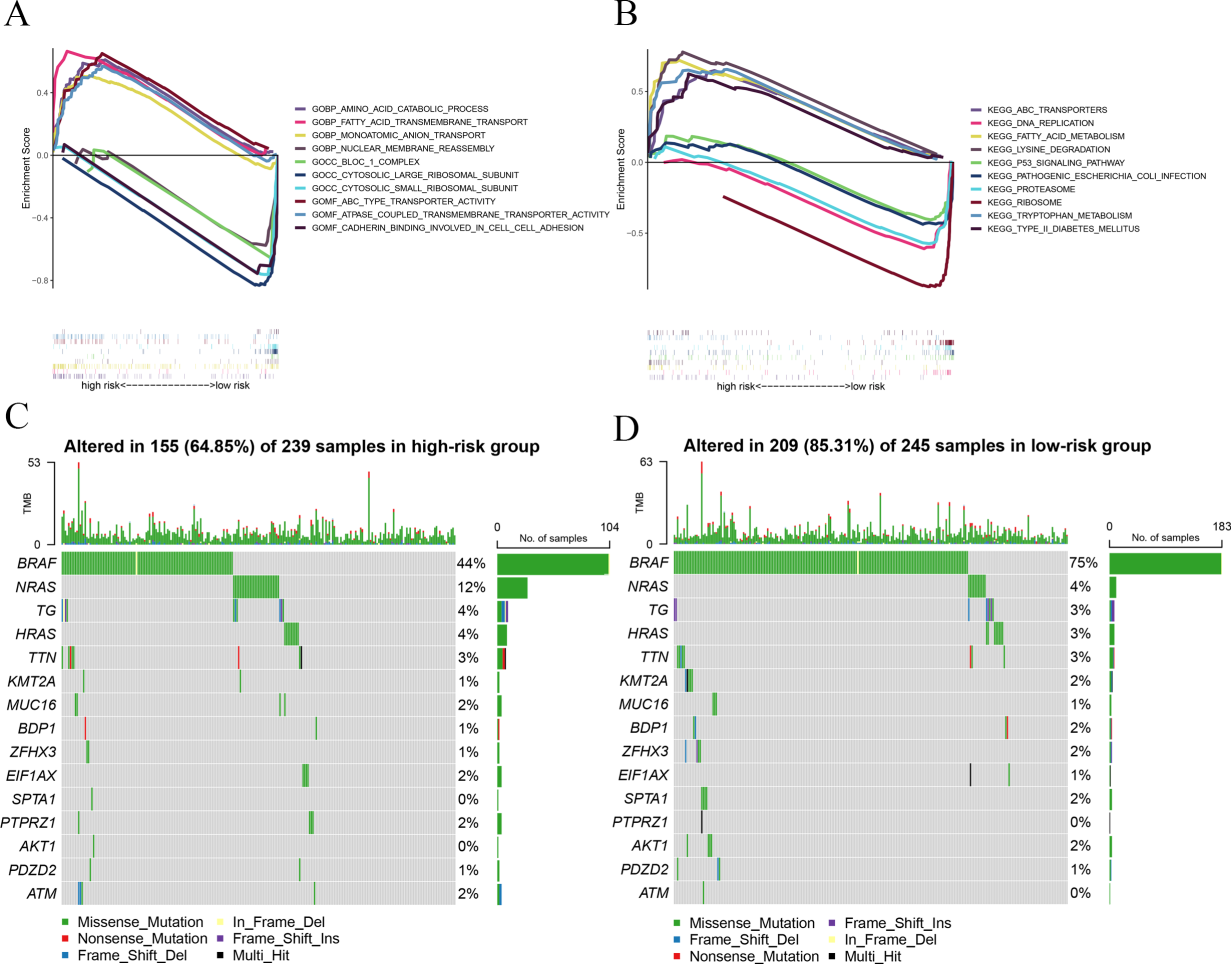
GSEA and mutation analysis. **(A)** GSEA of GO revealed the function of the prognostic signature. **(B)** GSEA of KEGG showed the potential biological pathway of the prognostic signature. **(C)** Mutation landscape displayed distinct genetic alterations in the high-risk group. **(D)** Mutation landscape verified distinct genetic alterations in the low-risk group.

All PTC samples were categorized into high and low groups according to the median expression levels of the hub genes. Next, we performed GSEA to explore the potential functional enrichment of the hub genes. Through GSEA of Gene Ontology (GO), we observed that the high-risk group was related to the AMINO ACID CATABOLIC PROCESS (BP), MONOATOMIC ANION TRANSPORT (BP), FATTY ACID TRANSMEMBRANE TRANSPORT (BP), ABC TYPE TRANSPORTER ACTIVITY (MF), and ATPASE COUPLED TRANSMEMBRANE TRANSPORTER ACTIVITY (MF), and the low-risk group was related to CYTOSOLIC LARGE RIBOSOMAL SUBUNIT (CC), NUCLEAR MEMBRANE REASSEMBLY (BP), BLOC 1 COMPLEX (CC), CYTOSOLIC SMALL RIBOSOMAL SUBUNIT (CC), and CADHERIN BINDING INVOLVED IN CELL-CELL ADHESION (MF) (**Figure 3A**). Through GSEA of KEGG, we observed that the high-risk group was associated with LYSINE DEGRADATION, ABC TRANSPORTERS, FATTY ACID METABOLISM, TYPE II DIABETES MELLITUS, and TRYPTOPHAN METABOLISM, and the low-risk group was associated with RIBOSOME, DNA REPLICATION, PROTEASOME, PATHOGENIC ESCHERICHIA COLI INFECTION, and P53 SIGNALING PATHWAY (**Figure 3B**).

To investigate the relationship between risk score and gene mutations, we analyzed the heterogeneity of gene mutations between the high-risk and low-risk groups. Compared with the high-risk group (64.85%), we uncovered that the low-risk group possessed a higher gene mutation ratio (85.31%) (**Figures 3C, D**). Furthermore, we also observed that *BRAF* and *NRAS* gene mutations are the most significant in those genes. The mutation ratio of *BRAF* is 75% in the low-risk group and 44% in the high-risk group. The mutation ratio of *NRAS* is 4% in the low-risk group and 12% in the high-risk group.

### Immune-related analysis of the prognostic signature

**Figure 4:**
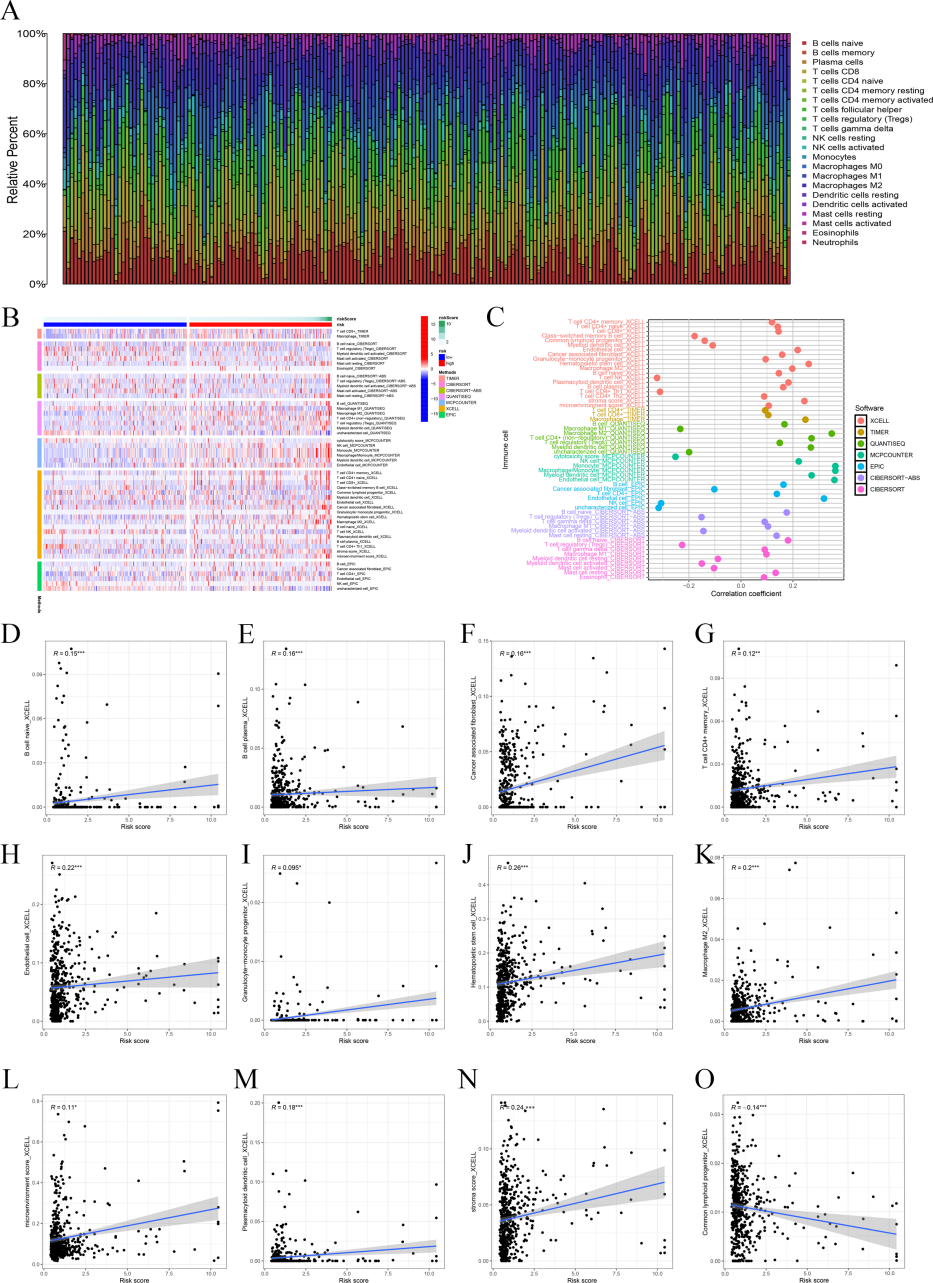
Immune-related analysis. **(A)** The ratio of 22 immune cells based on all PTC patients. **(B)** The distribution of various immune cells across samples with different risk scores. **(C)** Immune cell association analysis using seven algorithms revealed that risk score is closely associated with immune cells. (**D–O**) Correlation analysis of immune cells and risk scores.

In order to observe the immune condition of PTC patients and explore the relationship between FAM-related genes and tumor immunity, the CIBERSORT algorithm was employed to visualize the immune landscape of all PTC patients in the TCGA cohorts. The immune cell ratio of PTC patients was displayed in a heatmap (**Figure 4A**). The correlation results provided a comprehensive overview of the associations between risk scores and immune cells and stromal cells. We also employed a heatmap to visualize the distribution of various immune cells across samples with different risk scores (**Figure 4B**). Next, based on seven algorithms, we evaluated the association between immune cells and risk score (**Figure 4C**). We also showed the correlation between immune cells and risk score. The results showed that risk score is closely associated with multiple immune cells (**Figures 4D–O**) based on the xCell algorithm.

**Figure 5:**
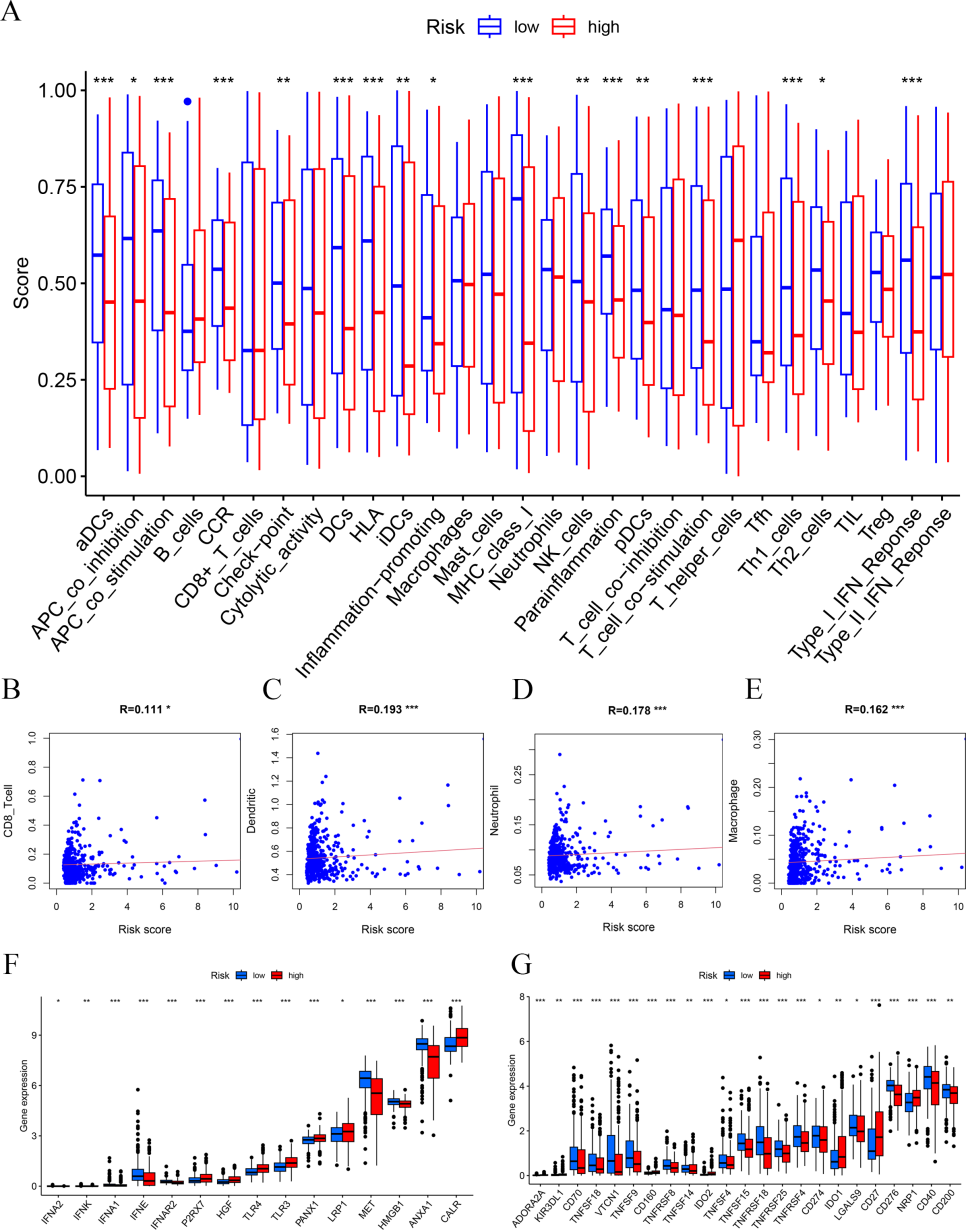
Immune function and immune cell association. **(A)** Immune function analysis-related risk score is related to potential functional immune cells. **(B)** The association with CD8 T cells. **(C)** The association with dendritic cells. **(D)** The association with neutrophils. **(E)** The association with macrophages. **(F)** Association between immune subgroups and ICD modulators. **(G)** Association between immune subgroups and ICP modulators. *p < 0.05, **p < 0.01, ***p < 0.001.

Furthermore, we used boxplots to illustrate the changes in immune cell abundance between the two groups, which revealed that 17 immune cell types were present in lower quantities in the high-risk group compared to the low-risk group (**Figure 5A**). Based on the TIMER algorithm, we specifically analyzed the relationship between six immune cells and risk scores, which showed a positive correlation for all six immune cell types (**Figures 5B–E**).

Immune checkpoints and immunogenic cell death (ICD)-related genes showed significant potential in tumor treatment, offering promising avenues to enhance antitumor immunity and improve therapeutic outcomes (27–29). We analyzed the expression differences of ICD-related genes and immune checkpoints between the high-risk and low-risk groups and observed that most immune checkpoints were upregulated in the low-risk group (**Figures 5F, G**). This suggests that, unlike the high-risk group, PTC patients in the low-risk group are more likely to respond favorably to immunotherapy.

### Silencing *SCD* inhibits PTC progression

**Figure 6:**
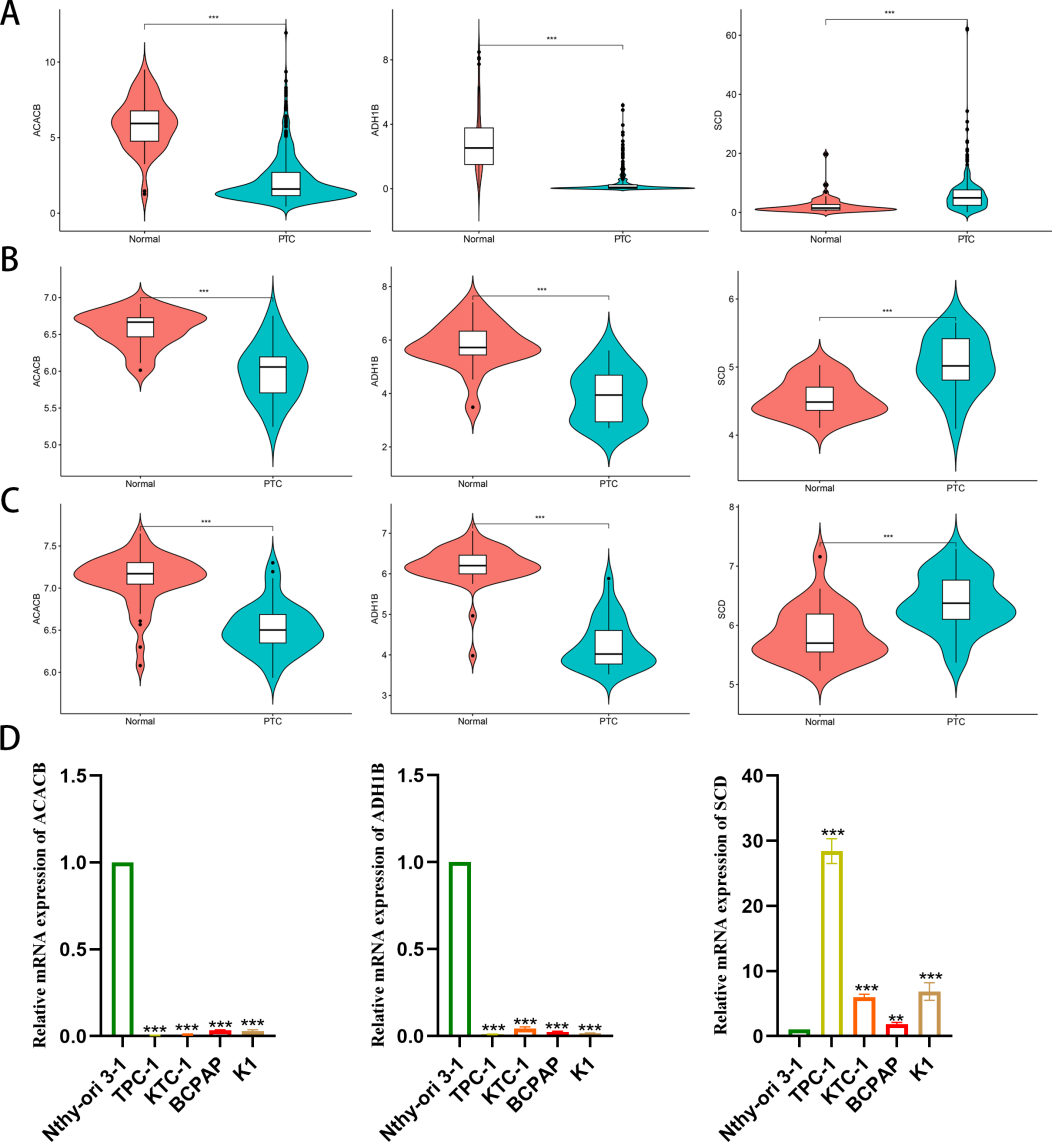
Differential expression analysis of the three FAM-related genes. **(A)** TCGA; **(B)** GSE29265; **(C)** GSE33630; **(D)** qPCR verified mRNA expression in cell lines. **p < 0.01, ***p < 0.001.

**Figure 7:**
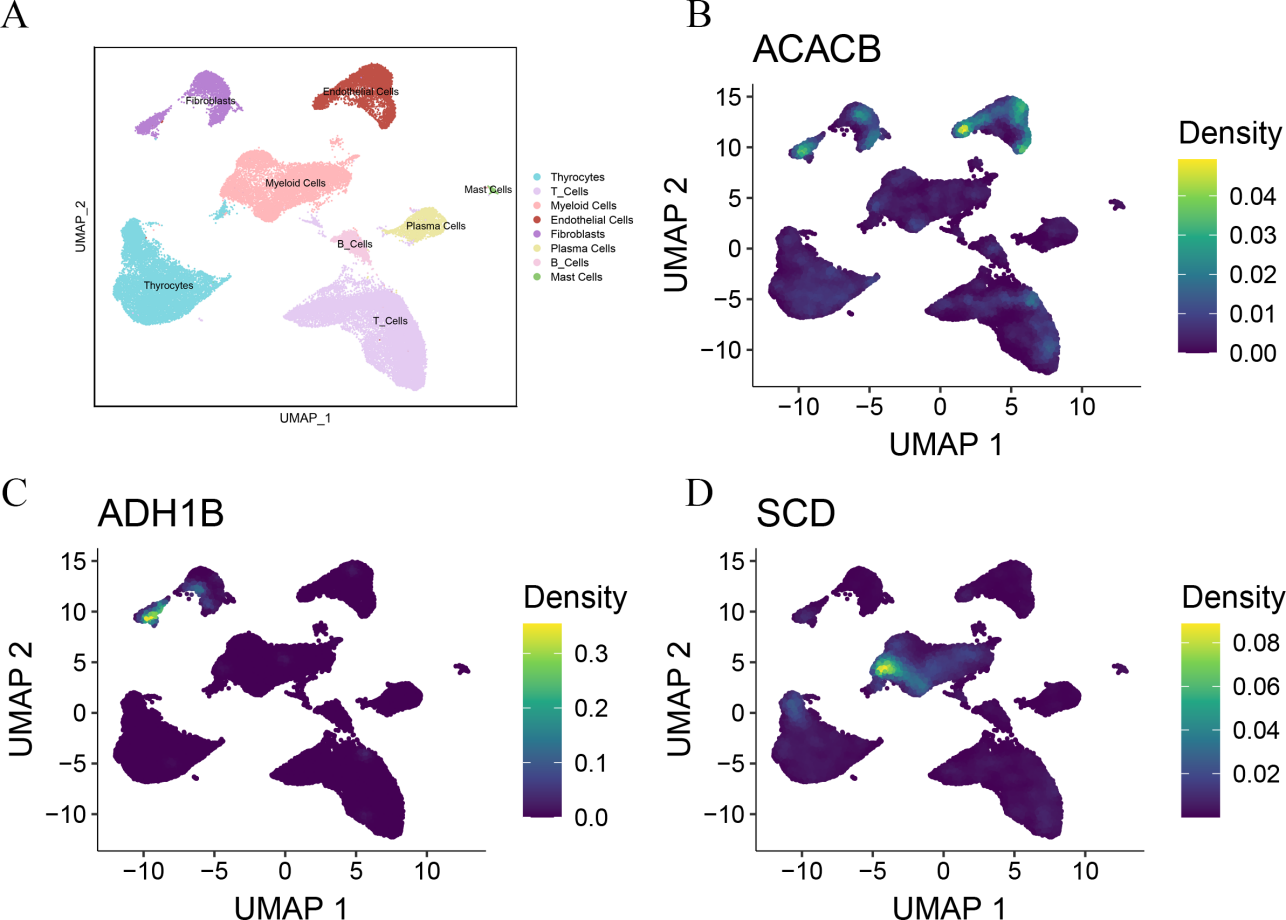
Single-cell analysis verified the expression of the three FAM-related genes. **(A)** UMAP plots of the major cell populations from PTC patients. Each point depicts a single cell, colored according to cell type. **(B)** The expression of *ACACB* in different cell types. **(C)** The expression of *ADH1B* in different cell types. **(D)** The expression of *SCD* in different cell types.

**Figure 8:**
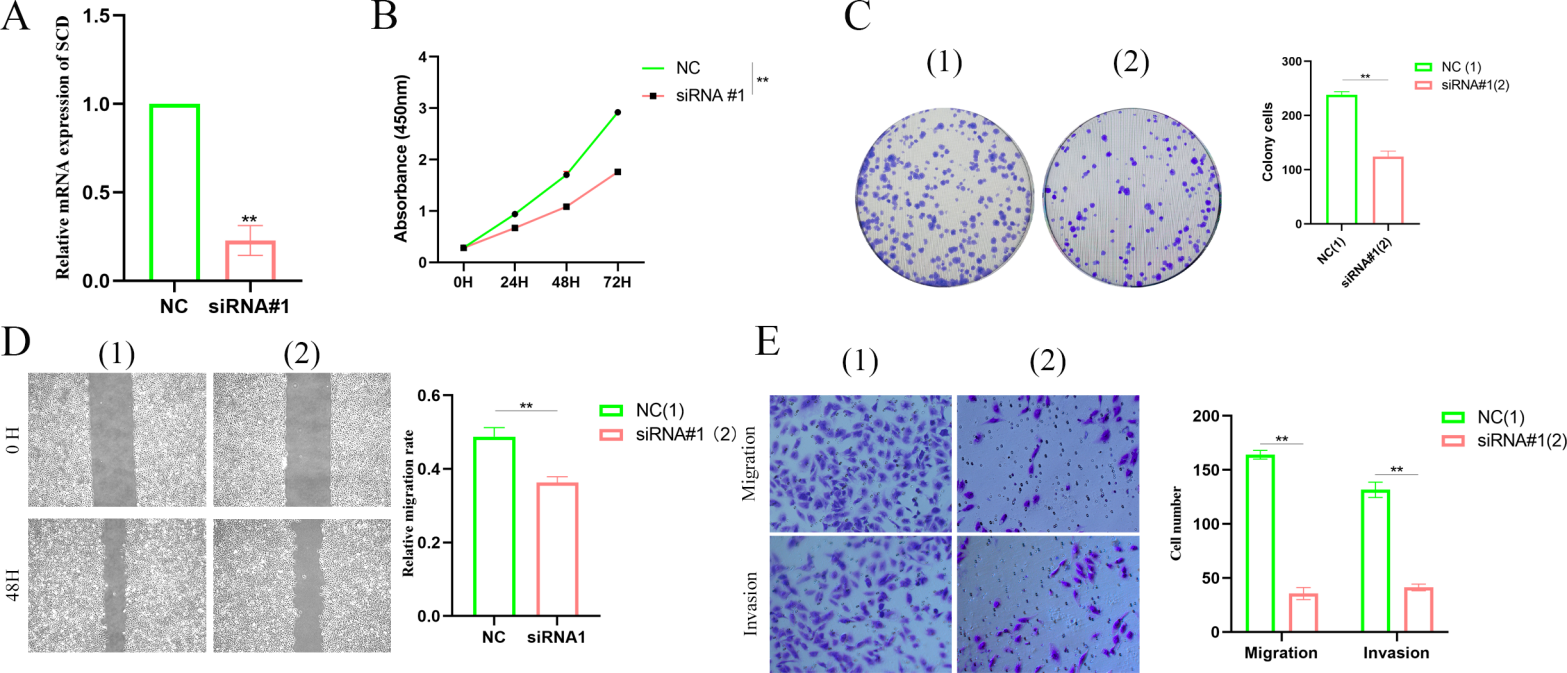
Silencing *SCD* inhibited PTC proliferation, migration, and invasion. **(A)** Silencing *SCD* downregulated *SCD* mRNA expression. **(B)** The CCK8 assay showed that silencing *SCD* inhibited PTC cell proliferation. **(C)** The colony formation assay depicted that silencing *SCD* disturbed PTC cell colony formation ability. **(D)** The scratch assay showed that silencing *SCD* controlled PTC cell migration. **(E)** The Transwell assay concluded that silencing *SCD* restrained PTC cell migration and invasion. **p < 0.01.

As a malignant tumor, DEGs may play an important role in PTC. Firstly, we analyzed the expression of three FAM-related genes across the TCGA, GSE29265, and GSE33630 cohorts. The results showed that, compared to PTC tissues, *ACACB* and *ADH1B* were more highly expressed in normal thyroid tissues, whereas *SCD* exhibited higher expression in PTC tissues (**Figures 6A–C**). Next, we used qPCR experiments to verify the expression of three FAM-related genes in normal thyroid cell lines and PTC cell lines. The results were consistent with the findings from the above bioinformatics analysis (**Figure 6D**). Moreover, using single-cell cohorts of PTC, we validated the relative expression levels of key genes across different cell types (**Figure 7A**). Our analysis revealed that *ACACB* is highly expressed in fibroblasts and endothelial cells (**Figure 7B**), *ADH1B* shows elevated expression in fibroblasts (**Figure 7C**), and *SCD* is predominantly expressed in myeloid cells (**Figure 7D**). Given that *SCD* is the only highly expressed gene among the three FAM-related genes, we plan to further investigate its role in PTC cells. We used siRNA to downregulate *SCD* expression in TPC-1 cells. Through the qPCR assay, *SCD* expression is significantly downregulated after using siRNA (**Figure 8A**). The CCK8 assay revealed that silencing *SCD* expression decreased the cell proliferation activity in the TPC-1 cell line (**Figure 8B**). The colony formation assay uncovered that colony formation ability was downregulated after silencing *SCD* expression (**Figure 8C**). Scratch assays observed that silencing *SCD* expression inhibited TPC-1 cell migration (**Figure 8D**). Transwell assays showed that silencing *SCD* expression suppressed TPC-1 cell migration and invasion (**Figure 8E**). Therefore, we concluded that *SCD* is a new PTC biomarker and silencing *SCD* expression inhibits PTC progression.

## Discussion

Thyroid cancer ranks as the seventh most prevalent cancer worldwide, with its incidence rapidly increasing (3). As the most common type of thyroid cancer, PTC is primarily treated with surgery, often followed by radioactive iodine therapy (30). Although most PTC patients have a favorable prognosis, surgical treatment often imposes considerable economic and psychological burdens. Therefore, exploring the mechanisms underlying PTC development and progression and identifying potential treatment targets are crucial for improving the diagnosis, treatment, and prognosis of PTC patients. Our study seeks to explore the influence of FAM-related genes on the prognosis of PTC patients. By constructing a prognostic signature of FAM-related genes, we aim to predict clinical outcomes and explore its associations with clinical features, immunotherapy responses, and tumor progression.

Growing evidence suggests that metabolic dysregulation is a key determinant of tumor progression, immunotherapy resistance, recurrence, and metastasis (31). Aberrant lipid metabolism is a hallmark of various cancers, enabling rapid proliferation by driving excessive endogenous lipid production or enhanced uptake of external lipids, with cancer cells relying on FAM, alongside aerobic glycolysis and glutamine consumption, to meet their heightened energy demands (4, 32–34). FAM primarily influences the biological behavior of cancer cells through the formation of membrane lipid constituents, as well as the generation and deposit of energy (5). The composition of fatty acids (FAs) in the cell membrane enhances cell survival and reduces lipotoxicity (35). Tumor cells can acquire FAs exogenously or synthesize them endogenously through dysregulated lipogenesis. Alterations in FA levels or excessive lipid accumulation can disrupt homeostasis and exacerbate cellular stress (4). Thyroid cancer, as a malignant tumor, has been reported to be closely related to the biological functions of FAM (36). However, the molecular markers related to FAM in thyroid cancer remain insufficiently characterized. Recently, the construction of cancer prognostic signatures relying on specific gene sets has gained increasing recognition (37–40). A reliable prognostic model can help clinicians accurately classify patients into high-risk and low-risk groups, applying it to personalized treatment decisions. We constructed a significant prognostic signature relying on FAM-related genes, validated its clinical relevance, and evaluated the immune landscape.

Firstly, we retrieved 309 FAM-related genes from the MSigDB database. Next, we identified 24 DEGs to construct prognostic signatures. Next, we built a prognostic signature, which included three FAM-related genes, namely, *ACACB*, *ADH1B*, and *SCD*. Furthermore, we evaluated the association of the immune landscape. We observed that the low-risk group showed elevated immune cell levels and enhanced expression of immune checkpoints. Hot tumors respond well to immunotherapy due to the high activity of immune cells, while cold tumors are unresponsive to immunotherapy because of the lack of immune cell infiltration (41–43). Therefore, we can classify the low-risk group as hot tumors and the high-risk group as cold tumors, with low-risk PTC patients likely to benefit from immunotherapy, providing a new direction for clinical decision-making. Lastly, we checked the expression of the three genes in normal thyroid and PTC cell lines, finding that *ACACB* and *ADH1B* had a lower expression in PTC cell lines, while *SCD* exhibited a higher expression in PTC cell lines.

The ACC family influences tumor progression, with *ACACB* being an important member of this family (44, 45). *ACACB* has been reported in various diseases, including cancer (46), diabetic nephropathy (47), obesity (48), diabetes (49), and hepatic steatosis (50). Li K. et al. found that *ACACB* is highly expressed in laryngeal cancer tissues and is closely related to cancer staging and the degree of cellular differentiation in laryngeal cancer (51). Valvo V. et al. observed that *BRAFV600E* downregulates *ACACB* expression, thereby disrupting the regulation of lipid metabolism, which promotes *de-novo* lipogenesis and reduces fatty acid oxidation (FAO), synergistically contributing to vemurafenib resistance and increased tumor growth, suggesting that rescuing *ACACB* may represent a novel strategy to overcome resistance to BRAFV600E inhibitors in PTC and improve treatment outcomes for patients with refractory disease (52).

*ADH1B* is a member of the *ADH1* family and is involved in the conversion of some alcohol products to aldehydes (53). The expression of *ADH1B* in human adipocytes is regulated by metabolic conditions (54, 55). Specifically, a downregulation of *ADH1B* expression has been shown to impair adipocyte differentiation, suggesting its crucial role in maintaining normal adipogenesis and metabolic function (55). Zhou Y. et al. believed that *ADH1B* is a key gene associated with afatinib, whose downregulation correlates with poorer prognosis and immune microenvironment changes in HCC patients, and they suggested *ADH1B* as a value target, offering a significant method for developing novel therapies for HCC (56). Morales LD et al. found that *ADH1B* disturbed the metabolic activity of adipose tissue, and its expression is inhibited by obesity, which is associated with insulin-stimulated glucose uptake (57). Yin D. et al. found that *ADH1B* is one of the genes with upregulated or downregulated expression in both LUAD and LUSC (58). Liu TT et al. considered *ADH1B* as one of the important genes linking Hashimoto’s thyroiditis (HT) and PTC, with potential diagnostic value (59). *ADH1B* affects the metabolic functions of adipose tissue, tumor progression, and metabolic diseases such as obesity, which means that it may serve as a potential treatment target for PTC patients.

*SCD* is a lipid-modifying enzyme that is upregulated in various cancers, including ovarian cancer (60), breast cancer (61), gastric cancer (GC) (62), and HCC (63). *SCD* promotes tumor progression and resistance in lung cancer by activating the *EGFR*/*PI3K*/*AKT* signaling pathway (64). *SCD* is also involved in GC chemoresistance by regulating the stemness and chemoresistance of GC cells through *AKAP-8L*, suggesting that *SCD* may overcome chemoresistance in GC (65). Lingrand M. et al. found that *SCD* regulates the migration of breast cancer cells by producing oleic acid, thereby promoting tumor metastasis (66). Yu Y. et al. uncovered that inhibiting *SCD* expression or function selectively excludes colon cancer stem cells through apoptosis, primarily by suppressing the *Wnt* and *Notch* signaling pathways (67). *SCD* plays a critical role in lipid metabolism in PTC, promoting PTC malignant progression through collaboration with *METTL16* and *YTHDC2* (68).

*ACACB*, *ADH1B*, and *SCD*, as key genes related to FAM, play important roles in tumor progression in PTC and may serve as potential therapeutic targets in PTC patients. Lastly, we also found that silencing *SCD* inhibited PTC cell proliferation, migration, and invasion. In conclusion, we found that FAM-related genes are involved in PTC progression, with *SCD* emerging as a potential therapeutic target. Further research is required to investigate its clinical implications and the underlying molecular mechanisms.

## Conclusion

In this study, we constructed a prognostic signature relying on FAM-related genes and validated its clinical applicability in PTC patients. We found that patients in the low-risk group represented higher immune cell infiltration and immune checkpoint expression, signifying that they are more likely to benefit from immunotherapy. Additionally, silencing *SCD* in PTC inhibited cell proliferation, migration, and invasion. Therefore, *SCD* could emerge as a promising biomarker for PTC and offers a potential treatment target for future interventions.

## Data Availability

The original contributions presented in the study are included in the article/**Supplementary Material**. Further inquiries can be directed to the corresponding author.
