# Supplementary material for: Fatty acid metabolism influences the immune microenvironment in papillary thyroid cancer and identifies SCD as a novel biomarker
Source: Front Endocrinol (Lausanne). 2025 Feb 27;16:1534393. doi: 10.3389/fendo.2025.1534393 (PMC11903245; doi:10.3389/fendo.2025.1534393)
Supplement: Supplementary file 1 [file Table1.docx]

**Supplementary File to**

**Fatty acid metabolism is related to the immune microenvironment changes of papillary thyroid cancer and SCD is a new tumor biomarker**

**Abbreviation**

**PTC, Papillary thyroid carcinoma; FAM, fatty acid metabolism; DEGs, Differentially expressed genes; FASN, fatty acid synthase; ACC, acetyl-CoA carboxylase; TCGA, The Cancer Genome Atlas; GEO, Gene Expression Omnibus; DMEM, Dulbecco's Modified Eagle Medium; DMEM/F12, Dulbecco's Modified Eagle Medium/Nutrient Mixture F-12; qPCR, quantitative Polymerase Chain Reaction; OD, optical density; cDNA, complementary DNA; PBS, Phosphate Buffered Saline; FAs, fatty acids; ACACB, Acetyl-CoA Carboxylase Beta; ADH1B, Alcohol Dehydrogenase 1B; FAO, fatty acid oxidation; HCC, hepatocellular carcinoma; LUAD, lung adenocarcinoma; LUSC, lung squamous cell carcinoma; HT, Hashimoto's thyroiditis; GC, gastric cancer.**

**Table S1. Primer sequence in this study.**

**Table S2. 309 FAM-related genes were originated from MSigDB database.**

**Table S3. 24 DEGs of 309 FAM-related genes.**

**Table S1.** **Primer sequence in this study.**

| **Primer name** | **Primer sequence** |
| --- | --- |
| β-actin (F)  β-actin (R)  ACACB (F)  ACACB (R)  ADH1B (F)  ADH1B (R)  SCD (F)  SCD (R) | CATGTACGTTGCTATCCAGGC  CTCCTTAATGTCACGCACGAT  CAAGCCGATCACCAAGAGTAAA  CCCTGAGTTATCAGAGGCTGG  AGGGGGCTGTTTATGGTGG  GGTACGGATACTTTTCCCAGAGT  TCTAGCTCCTATACCACCACCA  TCGTCTCCAACTTATCTCCTCC |

**Table S2. 309 FAM-related genes were originated from MSigDB database.**

| **Symbol** | **Description** |
| --- | --- |
| AADAT  ABCC1  ABCD1  ACAA1  ACAA2  ACACA  ACACB  ACAD10  ACAD11  ACADL  ACADM  ACADS  ACADSB  ACADVL  ACAT1  ACAT2  ACBD4  ACBD5  ACBD6  ACBD7  ACLY  ACO2  ACOT1  ACOT11  ACOT12  ACOT13  ACOT2  ACOT4  ACOT6  ACOT7  ACOT8  ACOT8  ACOT9  ACOX1  ACOX2  ACOX3  ACOXL  ACSBG1  ACSBG2  ACSF2  ACSF3  ACSL1  ACSL3  ACSL4  ACSL5  ACSL6  ACSM3  ACSM6  ACSS1  ADH1A  ADH1B  ADH1C  ADH4  ADH5  ADH6  ADH7  ADIPOR2  ADSL  AKR1C3  ALAD  ALDH1A1  ALDH1B1  ALDH2  ALDH3A1  ALDH3A2  ALDH9A1  ALDOA  ALOX12  ALOX12B  ALOX15  ALOX15B  ALOX5  ALOX5AP  ALOXE3  AMACR  AOC3  APEX1  AQP7  AUH  AWAT1  BCKDHB  BLVRA  BMPR1B  BPHL  CA2  CA4  CA6  CBR1  CBR1  CBR3  CBR4  CD1D  CD36  CEL  CIDEA  CPOX  CPT1A  CPT1B  CPT1C  CPT2  CROT  CRYZ  CYP1A1  CYP1A2  CYP1B1  CYP2C19  CYP2C8  CYP2C9  CYP2J2  CYP2U1  CYP4A11  CYP4A22  CYP4B1  CYP4F11  CYP4F2  CYP4F22  CYP4F3  CYP4F8  CYP8B1  D2HGDH  DBI  DECR1  DECR2  DHCR24  DLD  DLST  DPEP1  DPEP2  DPEP3  ECH1  ECHS1  ECI1  ECI2  EHHADH  ELOVL1  ELOVL2  ELOVL3  ELOVL4  ELOVL5  ELOVL6  ELOVL7  ENO2  ENO3  EPHX1  EPHX2  ERP29  ETFDH  FAAH  FAAH2  FABP1  FABP2  FADS1  FADS2  FASN  FH  FMO1  G0S2  GABARAPL1  GAD2  GAPDHS  GCDH  GGT1  GGT5  GLUL  GPD1  GPD2  GPX1  GPX2  GPX4  GRHPR  GSTZ1  H2AZ1  HACD1  HACD2  HACD3  HACD4  HACL1  HADH  HADHA  HADHB  HAO2  HCCS  HIBCH  HMGCL  HMGCS1  HMGCS2  HPGD  HPGDS  HSD17B10  HSD17B11  HSD17B12  HSD17B3  HSD17B4  HSD17B7  HSD17B8  HSDL2  HSP90AA1  HSPH1  HTD2  IDH1  IDH3B  IDH3G  IDI1  IL4I1  INMT  KMT5A  LDHA  LGALS1  LTA4H  LTC4S  MAOA  MAPKAPK2  MCAT  MCEE  MDH1  MDH2  ME1  MECR  METAP1  MGLL  MID1IP1  MIF  MIX23  MLYCD  MMAA  MMUT  MORC2  NBN  NCAPH2  NDUFAB1  NSDHL  NTHL1  NUDT19  NUDT7  ODC1  OLAH  OSTC  PCBD1  PCCA  PCCB  PCTP  PDHA1  PDHB  PECR  PHYH  PLA2G4A  PON1  PON2  PON3  PPARA  PPARD  PPT1  PPT2  PRDX6  PRKAA2  PRKAB2  PRKAG2  PRXL2B  PSME1  PTGDS  PTGES  PTGES2  PTGES3  PTGIS  PTGR1  PTGR2  PTGS1  PTGS2  PTPRG  PTS  RAP1GDS1  RDH11  RDH16  REEP6  RETSAT  RXRA  S100A10  SCD  SCD5  SCP2  SDHA  SDHC  SDHD  SERINC1  SLC22A5  SLC22A5  SLC25A17  SLC25A20  SLC27A2  SLC27A3  SMS  SUCLA2  SUCLG1  SUCLG2  TBXAS1  TDO2  TECR  TECRL  THEM4  THEM5  THRSP  TP53INP2  UBE2L6  UGDH  UROD  UROS  VNN1  XIST  YWHAH | Aminoadipate Aminotransferase  ATP Binding Cassette Subfamily C Member 1 (ABCC1 Blood Group)  ATP Binding Cassette Subfamily D Member 1  Acetyl-CoA Acyltransferase 1  Acetyl-CoA Acyltransferase 2  Acetyl-CoA Carboxylase Alpha  Acetyl-CoA Carboxylase Beta  Acyl-CoA Dehydrogenase Family Member 10  Acyl-CoA Dehydrogenase Family Member 11  Acyl-CoA Dehydrogenase Long Chain  Acyl-CoA Dehydrogenase Medium Chain  Acyl-CoA Dehydrogenase Short Chain  Acyl-CoA Dehydrogenase Short/Branched Chain  Acyl-CoA Dehydrogenase Very Long Chain  Acetyl-CoA Acetyltransferase 1  Acetyl-CoA Acetyltransferase 2  Acyl-CoA Binding Domain Containing 4  Acyl-CoA Binding Domain Containing 5  Acyl-CoA Binding Domain Containing 6  Acyl-CoA Binding Domain Containing 7  ATP Citrate Lyase  Aconitase 2  Acyl-CoA Thioesterase 1  Acyl-CoA Thioesterase 11  Acyl-CoA Thioesterase 12  Acyl-CoA Thioesterase 13  Acyl-CoA Thioesterase 2  Acyl-CoA Thioesterase 4  Acyl-CoA Thioesterase 6  Acyl-CoA Thioesterase 7  Acyl-CoA Thioesterase 8  Acyl-CoA Thioesterase 8  Acyl-CoA Thioesterase 9  Acyl-CoA Oxidase 1  Acyl-CoA Oxidase 2  Acyl-CoA Oxidase 3  Acyl-CoA Oxidase Like  Acyl-CoA Synthetase Bubblegum Family Member 1  Acyl-CoA Synthetase Bubblegum Family Member 2  Acyl-CoA Synthetase Family Member 2  Acyl-CoA Synthetase Family Member 3  Acyl-CoA Synthetase Long Chain Family Member 1  Acyl-CoA Synthetase Long Chain Family Member 3  Acyl-CoA Synthetase Long Chain Family Member 4  Acyl-CoA Synthetase Long Chain Family Member 5  Acyl-CoA Synthetase Long Chain Family Member 6  Acyl-CoA Synthetase Medium Chain Family Member 3  Acyl-CoA Synthetase Medium Chain Family Member 6  Acyl-CoA Synthetase Short Chain Family Member 1  Alcohol Dehydrogenase 1A (Class I), Alpha Polypeptide  Alcohol Dehydrogenase 1B (Class I), Beta Polypeptide  Alcohol Dehydrogenase 1C (Class I), Gamma Polypeptide  Alcohol Dehydrogenase 4 (Class II), Pi Polypeptide  Alcohol Dehydrogenase 5 (Class III), Chi Polypeptide  Alcohol Dehydrogenase 6 (Class V)  Alcohol Dehydrogenase 7 (Class IV), Mu Or Sigma Polypeptide  Adiponectin Receptor 2  Adenylosuccinate Lyase  Aldo-Keto Reductase Family 1 Member C3  Aminolevulinate Dehydratase  Aldehyde Dehydrogenase 1 Family Member A1  Aldehyde Dehydrogenase 1 Family Member B1  Aldehyde Dehydrogenase 2 Family Member  Aldehyde Dehydrogenase 3 Family Member A1  Aldehyde Dehydrogenase 3 Family Member A2  Aldehyde Dehydrogenase 9 Family Member A1  Aldolase, Fructose-Bisphosphate A  Arachidonate 12-Lipoxygenase, 12S Type  Arachidonate 12-Lipoxygenase, 12R Type  Arachidonate 15-Lipoxygenase  Arachidonate 15-Lipoxygenase Type B  Arachidonate 5-Lipoxygenase  Arachidonate 5-Lipoxygenase Activating Protein  Arachidonate Epidermal Lipoxygenase 3  Alpha-Methylacyl-CoA Racemase  Amine Oxidase Copper Containing 3  Apurinic/Apyrimidinic Endodeoxyribonuclease 1  Aquaporin 7  AU RNA Binding Methylglutaconyl-CoA Hydratase  Acyl-CoA Wax Alcohol Acyltransferase 1  Branched Chain Keto Acid Dehydrogenase E1 Subunit Beta  Biliverdin Reductase A  Bone Morphogenetic Protein Receptor Type 1B  Biphenyl Hydrolase Like  Carbonic Anhydrase 2  Carbonic Anhydrase 4  Carbonic Anhydrase 6  Carbonyl Reductase 1  Carbonyl Reductase 1  Carbonyl Reductase 3  Carbonyl Reductase 4  CD1d Molecule  CD36 Molecule (CD36 Blood Group)  Carboxyl Ester Lipase  Cell Death Inducing DFFA Like Effector A  Coproporphyrinogen Oxidase  Carnitine Palmitoyltransferase 1A  Carnitine Palmitoyltransferase 1B  Carnitine Palmitoyltransferase 1C  Carnitine Palmitoyltransferase 2  Carnitine O-Octanoyltransferase  Crystallin Zeta  Cytochrome P450 Family 1 Subfamily A Member 1  Cytochrome P450 Family 1 Subfamily A Member 2  Cytochrome P450 Family 1 Subfamily B Member 1  Cytochrome P450 Family 2 Subfamily C Member 19  Cytochrome P450 Family 2 Subfamily C Member 8  Cytochrome P450 Family 2 Subfamily C Member 9  Cytochrome P450 Family 2 Subfamily J Member 2  Cytochrome P450 Family 2 Subfamily U Member 1  Cytochrome P450 Family 4 Subfamily A Member 11  Cytochrome P450 Family 4 Subfamily A Member 22  Cytochrome P450 Family 4 Subfamily B Member 1  Cytochrome P450 Family 4 Subfamily F Member 11  Cytochrome P450 Family 4 Subfamily F Member 2  Cytochrome P450 Family 4 Subfamily F Member 22  Cytochrome P450 Family 4 Subfamily F Member 3  Cytochrome P450 Family 4 Subfamily F Member 8  Cytochrome P450 Family 8 Subfamily B Member 1  D-2-Hydroxyglutarate Dehydrogenase  Diazepam Binding Inhibitor, Acyl-CoA Binding Protein  2,4-Dienoyl-CoA Reductase 1  2,4-Dienoyl-CoA Reductase 2  24-Dehydrocholesterol Reductase  Dihydrolipoamide Dehydrogenase  Dihydrolipoamide S-Succinyltransferase  Dipeptidase 1  Dipeptidase 2  Dipeptidase 3  Enoyl-CoA Hydratase 1  Enoyl-CoA Hydratase, Short Chain 1  Enoyl-CoA Delta Isomerase 1  Enoyl-CoA Delta Isomerase 2  Enoyl-CoA Hydratase And 3-Hydroxyacyl CoA Dehydrogenase  ELOVL Fatty Acid Elongase 1  ELOVL Fatty Acid Elongase 2  ELOVL Fatty Acid Elongase 3  ELOVL Fatty Acid Elongase 4  ELOVL Fatty Acid Elongase 5  ELOVL Fatty Acid Elongase 6  ELOVL Fatty Acid Elongase 7  Enolase 2  Enolase 2  Epoxide Hydrolase 1  Epoxide Hydrolase 2  Endoplasmic Reticulum Protein 29  Electron Transfer Flavoprotein Dehydrogenase  Fatty Acid Amide Hydrolase  Fatty Acid Amide Hydrolase 2  Fatty Acid Binding Protein 1  Fatty Acid Binding Protein 2  Fatty Acid Desaturase 1  Fatty Acid Desaturase 2  Fatty Acid Synthase  Fumarate Hydratase  Flavin Containing Dimethylaniline Monoxygenase 1  G0/G1 Switch 2  GABA Type A Receptor Associated Protein Like 1  Glutamate Decarboxylase 2  Glyceraldehyde-3-Phosphate Dehydrogenase, Spermatogenic  Glutaryl-CoA Dehydrogenase  Gamma-Glutamyltransferase 1  Gamma-Glutamyltransferase 5  Glutamate-Ammonia Ligase  Glycerol-3-Phosphate Dehydrogenase 1  Glycerol-3-Phosphate Dehydrogenase 2  Glutathione Peroxidase 1  Glutathione Peroxidase 2  Glutathione Peroxidase 4  Glyoxylate And Hydroxypyruvate Reductase  Glutathione S-Transferase Zeta 1  H2A.Z Variant Histone 1  3-Hydroxyacyl-CoA Dehydratase 1  3-Hydroxyacyl-CoA Dehydratase 2  3-Hydroxyacyl-CoA Dehydratase 3  3-Hydroxyacyl-CoA Dehydratase 4  2-Hydroxyacyl-CoA Lyase 1  Hydroxyacyl-CoA Dehydrogenase  Hydroxyacyl-CoA Dehydrogenase Trifunctional Multienzyme Complex Subunit Alpha  Hydroxyacyl-CoA Dehydrogenase Trifunctional Multienzyme Complex Subunit Beta  Hydroxyacid Oxidase 2  Holocytochrome C Synthase  3-Hydroxyisobutyryl-CoA Hydrolase  3-Hydroxy-3-Methylglutaryl-CoA Lyase  3-Hydroxy-3-Methylglutaryl-CoA Synthase 1  3-Hydroxy-3-Methylglutaryl-CoA Synthase 2  15-Hydroxyprostaglandin Dehydrogenase  Hematopoietic Prostaglandin D Synthase  Hydroxysteroid 17-Beta Dehydrogenase 10  Hydroxysteroid 17-Beta Dehydrogenase 11  Hydroxysteroid 17-Beta Dehydrogenase 12  Hydroxysteroid 17-Beta Dehydrogenase 3  Hydroxysteroid 17-Beta Dehydrogenase 4  Hydroxysteroid 17-Beta Dehydrogenase 7  Hydroxysteroid 17-Beta Dehydrogenase 8  Hydroxysteroid Dehydrogenase Like 2  Heat Shock Protein 90 Alpha Family Class A Member 1  Heat Shock Protein Family H (Hsp110) Member 1  Hydroxyacyl-Thioester Dehydratase Type 2  Isocitrate Dehydrogenase (NADP(+)) 1  Isocitrate Dehydrogenase (NAD(+)) 3 Non-Catalytic Subunit Beta  Isocitrate Dehydrogenase (NAD(+)) 3 Non-Catalytic Subunit Gamma  Isopentenyl-Diphosphate Delta Isomerase 1  Interleukin 4 Induced 1  Indolethylamine N-Methyltransferase  Lysine Methyltransferase 5A  Lactate Dehydrogenase A  Galectin 1  Leukotriene A4 Hydrolase  Leukotriene C4 Synthase  Monoamine Oxidase A  MAPK Activated Protein Kinase 2  Malonyl-CoA-Acyl Carrier Protein Transacylase  Methylmalonyl-CoA Epimerase  Malate Dehydrogenase 1  Malate Dehydrogenase 2  Malic Enzyme 1  Mitochondrial Trans-2-Enoyl-CoA Reductase  Methionyl Aminopeptidase 1  Monoglyceride Lipase  MID1 Interacting Protein 1  Macrophage Migration Inhibitory Factor  Mitochondrial Matrix Import Factor 23  Malonyl-CoA Decarboxylase  Metabolism Of Cobalamin Associated A  Methylmalonyl-CoA Mutase  MORC Family CW-Type Zinc Finger 2  Nibrin  Non-SMC Condensin II Complex Subunit H2  NADH:Ubiquinone Oxidoreductase Subunit AB1  NAD(P) Dependent Steroid Dehydrogenase-Like  Nth Like DNA Glycosylase 1  Nudix Hydrolase 19  Nudix Hydrolase 7  Ornithine Decarboxylase 1  Oleoyl-ACP Hydrolase  Oligosaccharyltransferase Complex Non-Catalytic Subunit  Pterin-4 Alpha-Carbinolamine Dehydratase 1  Propionyl-CoA Carboxylase Subunit Alpha  Propionyl-CoA Carboxylase Subunit Beta  Phosphatidylcholine Transfer Protein  Pyruvate Dehydrogenase E1 Subunit Alpha 1  Pyruvate Dehydrogenase E1 Subunit Beta  Peroxisomal Trans-2-Enoyl-CoA Reductase  Phytanoyl-CoA 2-Hydroxylase  Phospholipase A2 Group IVA  Paraoxonase 1  Paraoxonase 2  Paraoxonase 3  Peroxisome Proliferator Activated Receptor Alpha  Peroxisome Proliferator Activated Receptor Delta  Palmitoyl-Protein Thioesterase 1  Palmitoyl-Protein Thioesterase 2  Peroxiredoxin 6  Protein Kinase AMP-Activated Catalytic Subunit Alpha 2  Protein Kinase AMP-Activated Non-Catalytic Subunit Beta 2  Protein Kinase AMP-Activated Non-Catalytic Subunit Gamma 2  Peroxiredoxin Like 2B  Proteasome Activator Subunit 1  Prostaglandin D2 Synthase  Prostaglandin E Synthase  Prostaglandin E Synthase 2  Prostaglandin E Synthase 3  Prostaglandin I2 Synthase  Prostaglandin Reductase 1  Prostaglandin Reductase 2  Prostaglandin-Endoperoxide Synthase 1  Prostaglandin-Endoperoxide Synthase 2  Protein Tyrosine Phosphatase Receptor Type G  6-Pyruvoyltetrahydropterin Synthase  Rap1 GTPase-GDP Dissociation Stimulator 1  Retinol Dehydrogenase 11  Retinol Dehydrogenase 16  Receptor Accessory Protein 6  Retinol Saturase  Retinoid X Receptor Alpha  S100 Calcium Binding Protein A10  Stearoyl-CoA Desaturase  Stearoyl-CoA Desaturase 5  Sterol Carrier Protein 2  Succinate Dehydrogenase Complex Flavoprotein Subunit A  Succinate Dehydrogenase Complex Subunit C  Succinate Dehydrogenase Complex Subunit D  Serine Incorporator 1  Solute Carrier Family 22 Member 5  Solute Carrier Family 22 Member 5  Solute Carrier Family 25 Member 17  Solute Carrier Family 25 Member 20  Solute Carrier Family 27 Member 2  Solute Carrier Family 27 Member3  Spermine Synthase  Succinate-CoA Ligase ADP-Forming Subunit Beta  Succinate-CoA Ligase GDP/ADP-Forming Subunit Alpha  Succinate-CoA Ligase GDP-Forming Subunit Beta  Thromboxane A Synthase 1  Tryptophan 2,3-Dioxygenase  Trans-2,3-Enoyl-CoA Reductase  Trans-2,3-Enoyl-CoA Reductase Like  Thioesterase Superfamily Member 4  Thioesterase Superfamily Member 5  Thyroid Hormone Responsive  Tumor Protein P53 Inducible Nuclear Protein 2  Ubiquitin Conjugating Enzyme E2 L6  UDP-Glucose 6-Dehydrogenase  Uroporphyrinogen Decarboxylase  Uroporphyrinogen III Synthase  Vanin 1  X Inactive Specific Transcript  Tyrosine 3-Monooxygenase/Tryptophan 5-Monooxygenase Activation Protein Eta |

**Table S3. 24 DEGs of 309 FAM-related genes.**

| **Symbol** | **Description** |
| --- | --- |
| ACACB  ACBD7  ACOT7  ADH1B  ALDH1A1  ALOX15B  ALOX5  CA2  CA4  CPT1C  CYP1B1  CYP4B1  ELOVL4  FMO1  G0S2  HACD1  HPGDS  LGALS1  OLAH  S100A10  SCD  TDO2  THRSP  TP53INP2 | Acetyl-CoA Carboxylase Beta  Acyl-CoA Binding Domain Containing 7  Acyl-CoA Thioesterase 7  Alcohol Dehydrogenase 1B (Class I), Beta Polypeptide  Aldehyde Dehydrogenase 1 Family Member A1  Arachidonate 15-Lipoxygenase Type B  Arachidonate 5-Lipoxygenase  Carbonic Anhydrase 2  Carbonic Anhydrase 4  Carnitine Palmitoyltransferase 1C  Cytochrome P450 Family 1 Subfamily B Member 1  Cytochrome P450 Family 4 Subfamily B Member 1  ELOVL Fatty Acid Elongase 4  Flavin Containing Dimethylaniline Monoxygenase 1  G0/G1 Switch 2  3-Hydroxyacyl-CoA Dehydratase 1  Hematopoietic Prostaglandin D Synthase  Galectin 1  Oleoyl-ACP Hydrolase  S100 Calcium Binding Protein A10  Stearoyl-CoA Desaturase  Tryptophan 2,3-Dioxygenase  Thyroid Hormone Responsive  Tumor Protein P53 Inducible Nuclear Protein 2 |
